# Supplementary figures and images for: Structure of the human P2X3 receptor reveals the basis for subtype-selective inhibition by sivopixant
Source: PLoS Biol. 2026 Apr 22;24(4):e3003777. doi: 10.1371/journal.pbio.3003777 (PMC13132459; doi:10.1371/journal.pbio.3003777)

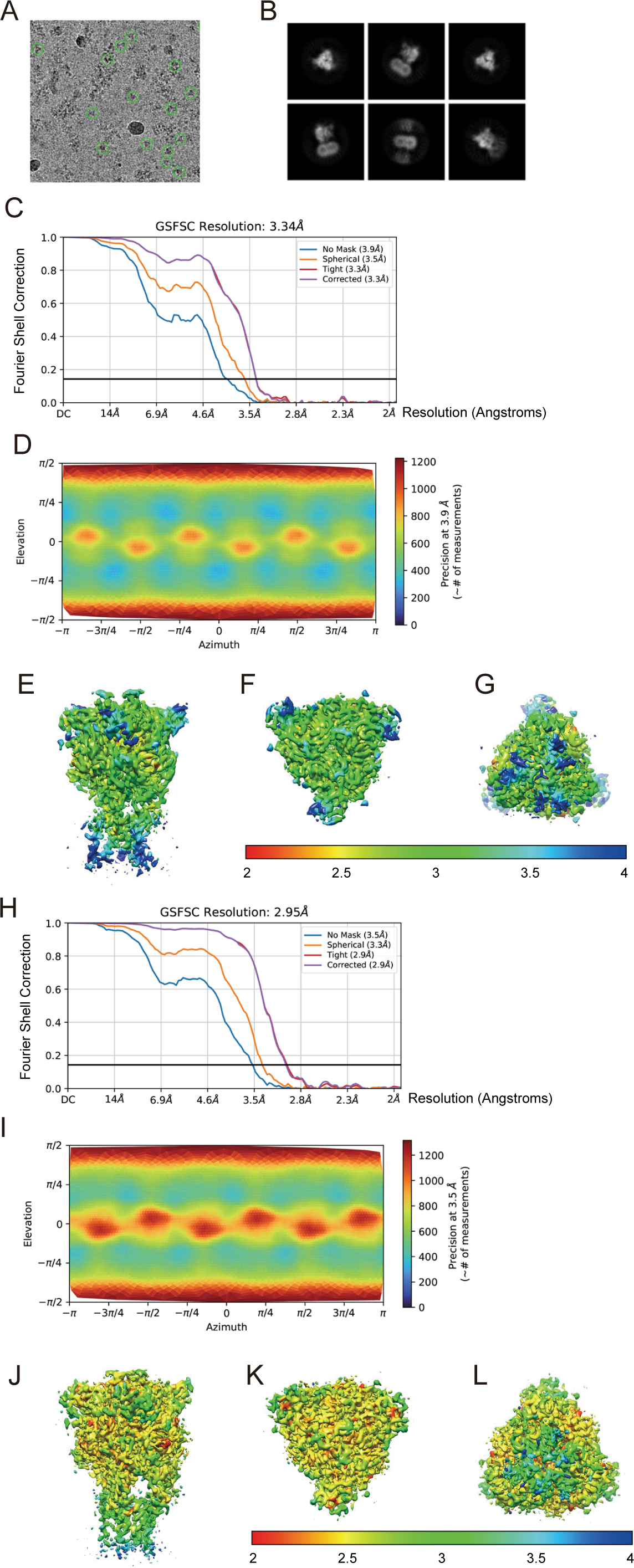

Supplement: S1 Fig — (A) Representative cryo-EM image of human P2X3 receptor particles. (B) Representative 2D class averages. (C–G) For the sivopixant- and ATP-bound human P2X3 receptor: (C) Gold-standard Fourier shell correlation (FSC) curves for resolution estimation. (D) Angular distribution of the particles used for the final map. (E–G) Side view (E), top view from the extracellular side (F), and bottom view from the cytoplasmic side (G) of the cryo-EM density map colored according to the local resolution, estimated using CryoSPARC. (H–L) For the ATP-bound human P2X3 receptor: (H) Gold-standard FSC curves for resolution estimation. (I) Angular distribution of the particles used for the final map. (J–L) Side view (J), top view from the extracellular side (K), and bottom view from the cytoplasmic side (L) of the cryo-EM density map colored according to the local resolution, estimated using CryoSPARC. (TIF) [file pbio.3003777.s001.tif]

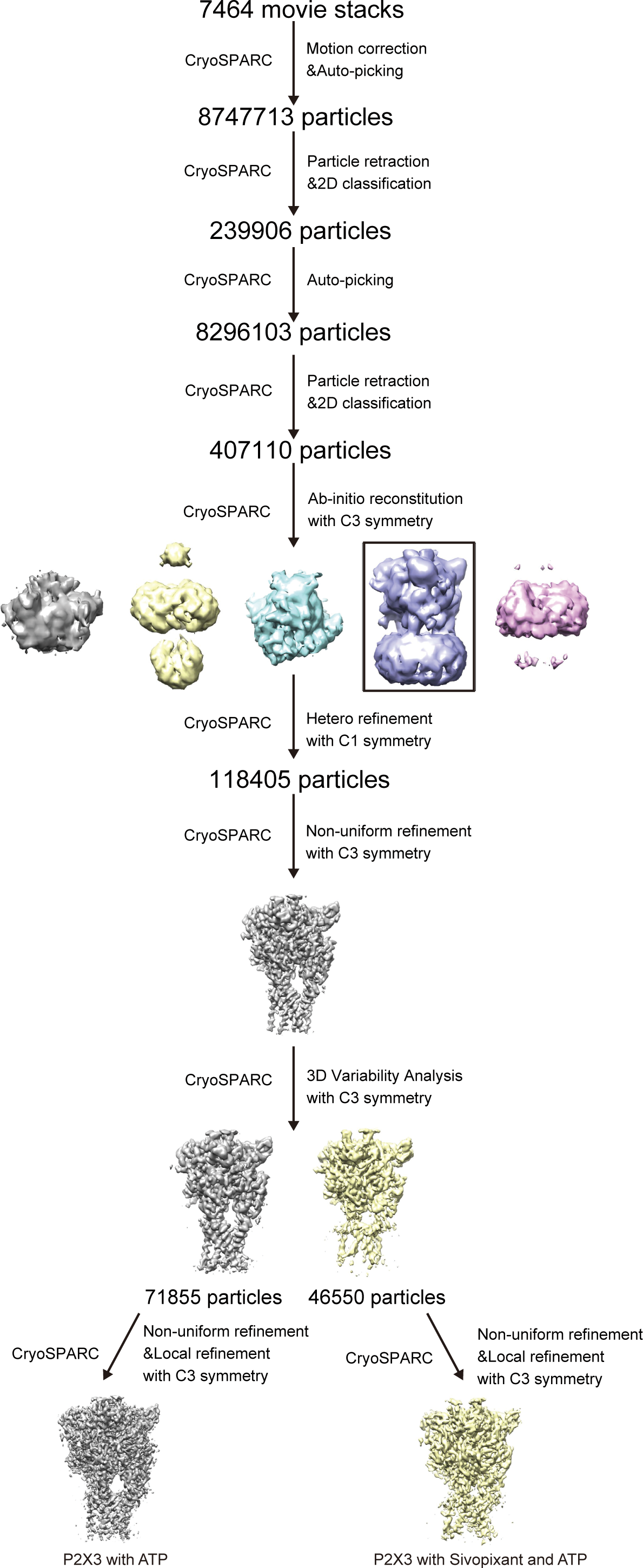

Supplement: S2 Fig — All the processing steps were performed using CryoSPARC. (TIF) [file pbio.3003777.s002.tif]

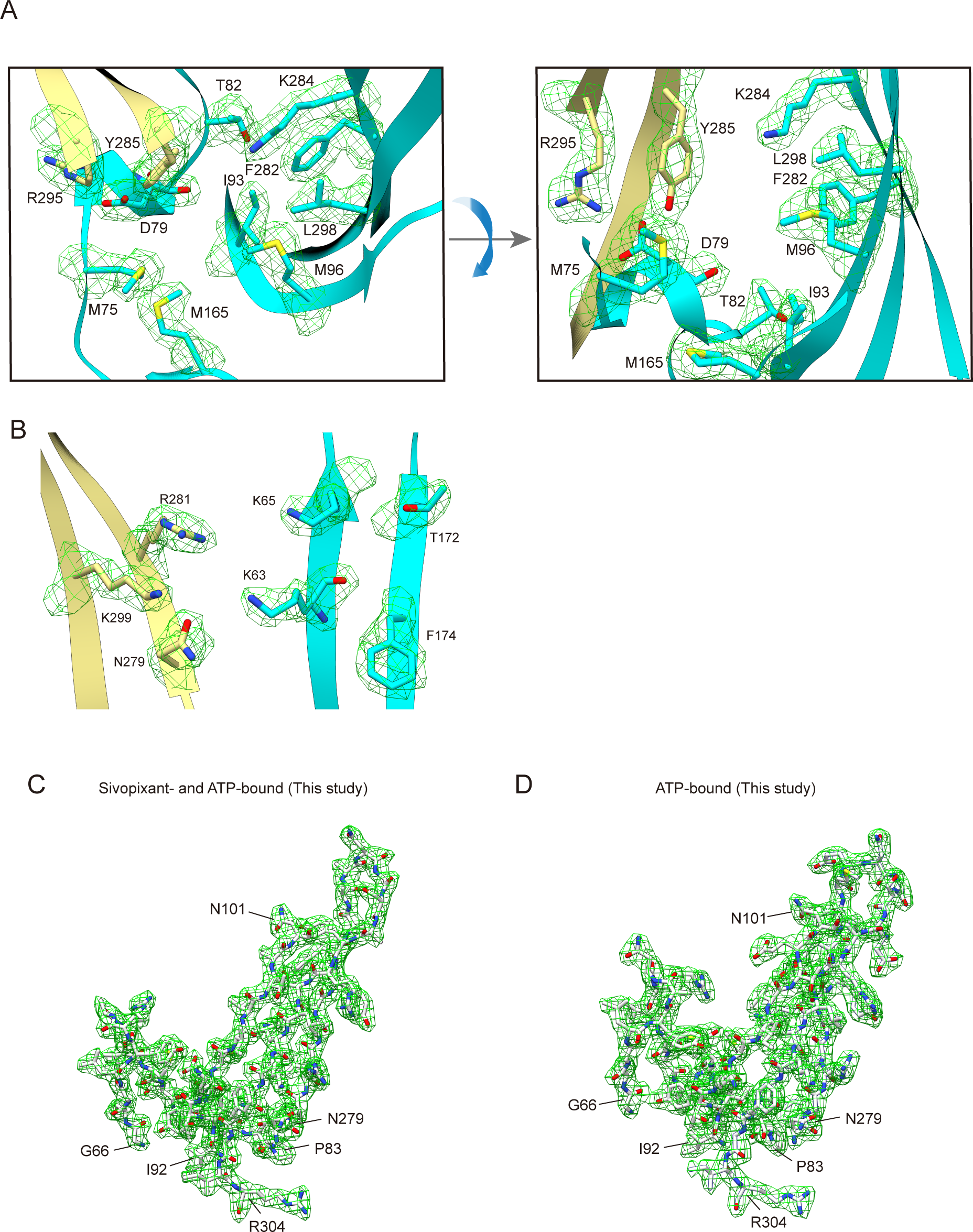

Supplement: S3 Fig — (A, B) Close-up views of the cryo-EM density map around residues lining the sivopixant (A)- and ATP (B)-binding site. (C, D) Cryo-EM density maps for residues in the upper body domain in the sivopixant- and the ATP-bound structure (C) and ATP-bound (D) hP2X3 receptor structures. Residues are shown as sticks, and the cryo-EM density is shown as a green mesh. (TIF) [file pbio.3003777.s003.tif]

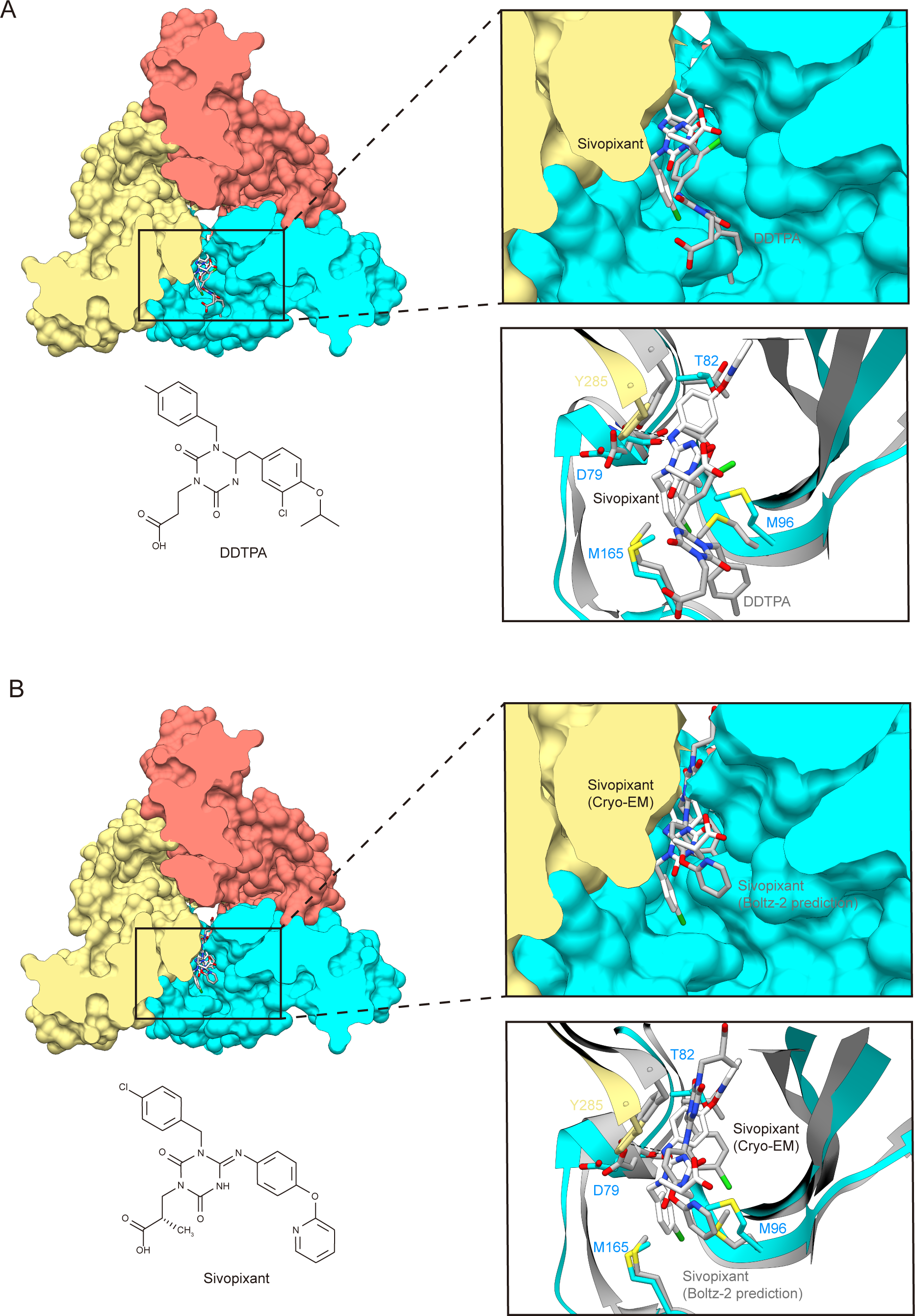

Supplement: S4 Fig — (A, B) Superimposition of the previously-predicted DDTPA-bound P2X3 receptor structure (A) and the Boltz-2-predicted sivopixant-bound P2X3 receptor structure (B) onto the cryo-EM structure of sivopixant- and ATP-bound P2X3 receptor viewed from the extracellular side. The cryo-EM structure is shown in surface representation (upper right panel) or in ribbon representation (lower right panel) together with the superposed predicted structures (gray). The confidence score, protein ipTM, ligand ipTM, and complex pLDDT predicted by Boltz-2 were 0.81, 0.84, 0.53, and 0.80, respectively. (TIF) [file pbio.3003777.s004.tif]

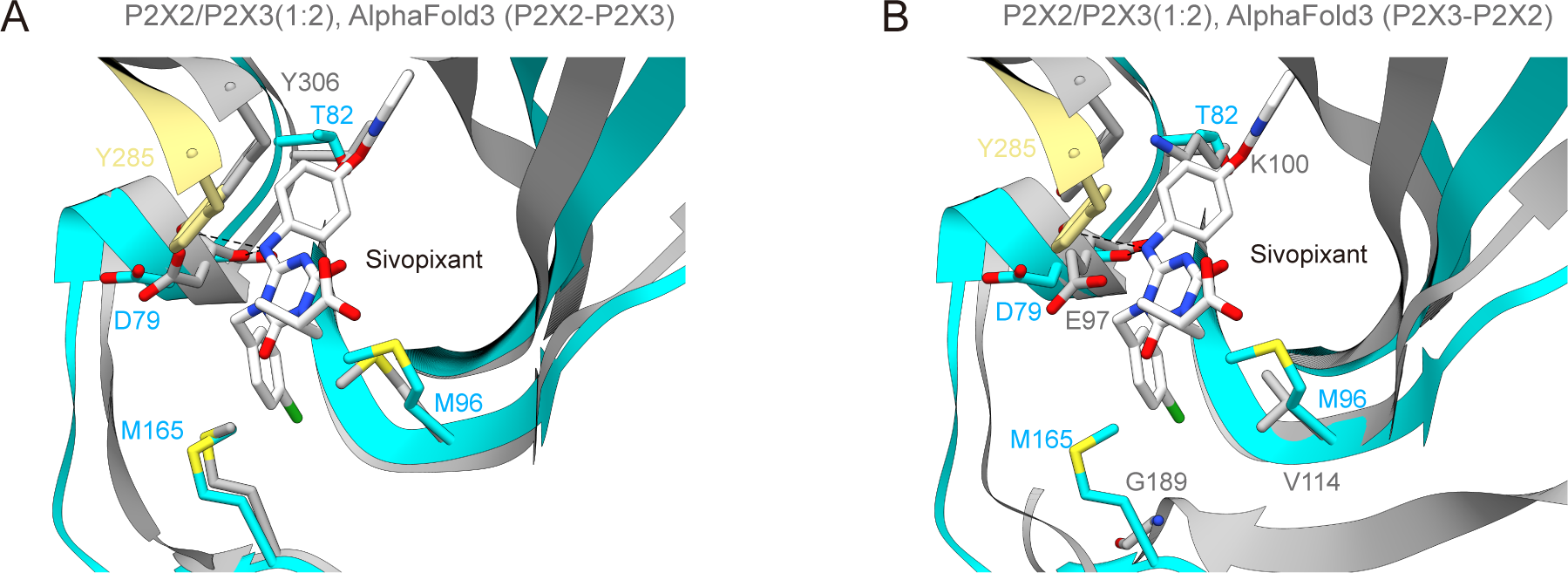

Supplement: S5 Fig — (A, B) The predicted heterotrimer structure formed by one P2X2 subunit and two P2X3 subunits (AlphaFold3, ipTM = 0.77) superposed onto the P2X3 receptor structure shown in gray. In A, the gray chain superposed onto the yellow chain is the P2X2 subunit, while the gray chain superposed onto the blue chain is the P2X3 subunit. In B, the gray chain superposed onto the yellow chain is the P2X3 subunit, while the gray chain superposed onto the blue chain is the P2X2 subunit. (TIF) [file pbio.3003777.s005.tif]

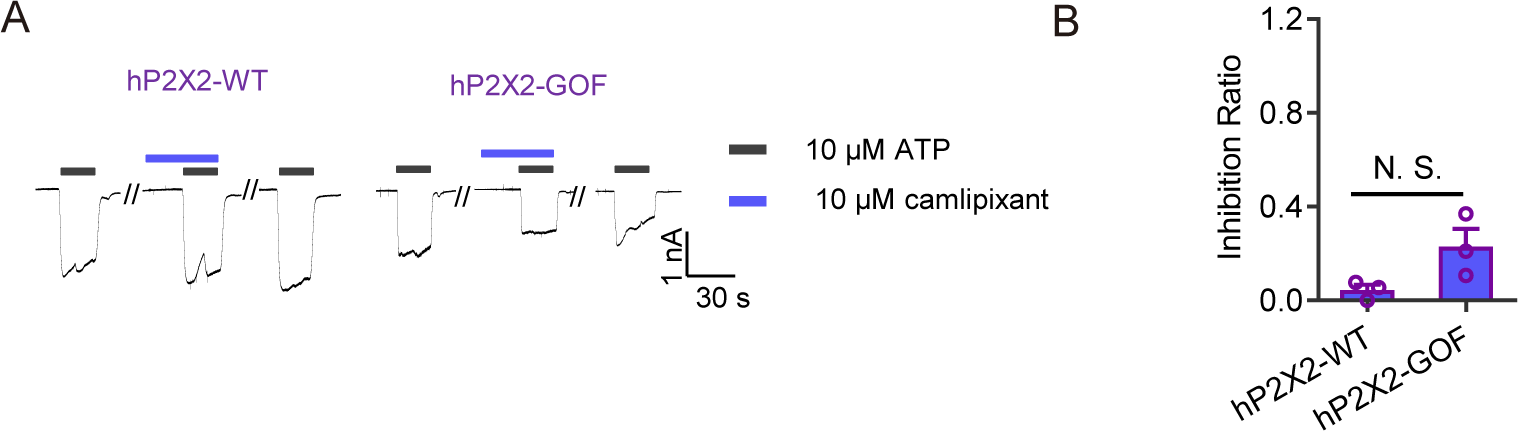

Supplement: S6 Fig — (A) Representative current traces showing the effects of camlipixant on ATP-evoked currents of human P2X2 wild-type (hP2X2-WT) and gain-of-function mutant (hP2X2-GOF) receptors. ATP and camlipixant were applied at 10 uM as indicated. (B) Inhibition ratios of camlipixant on ATP-evoked currents of hP2X2-WT and hP2X2-GOF receptors (mean ± SEM, n = 3–4). One-way ANOVA followed by Tukey’s multiple comparisons test, N.S., not significant. The data underlying this figure can be found in S1 Data. (TIF) [file pbio.3003777.s006.tif]

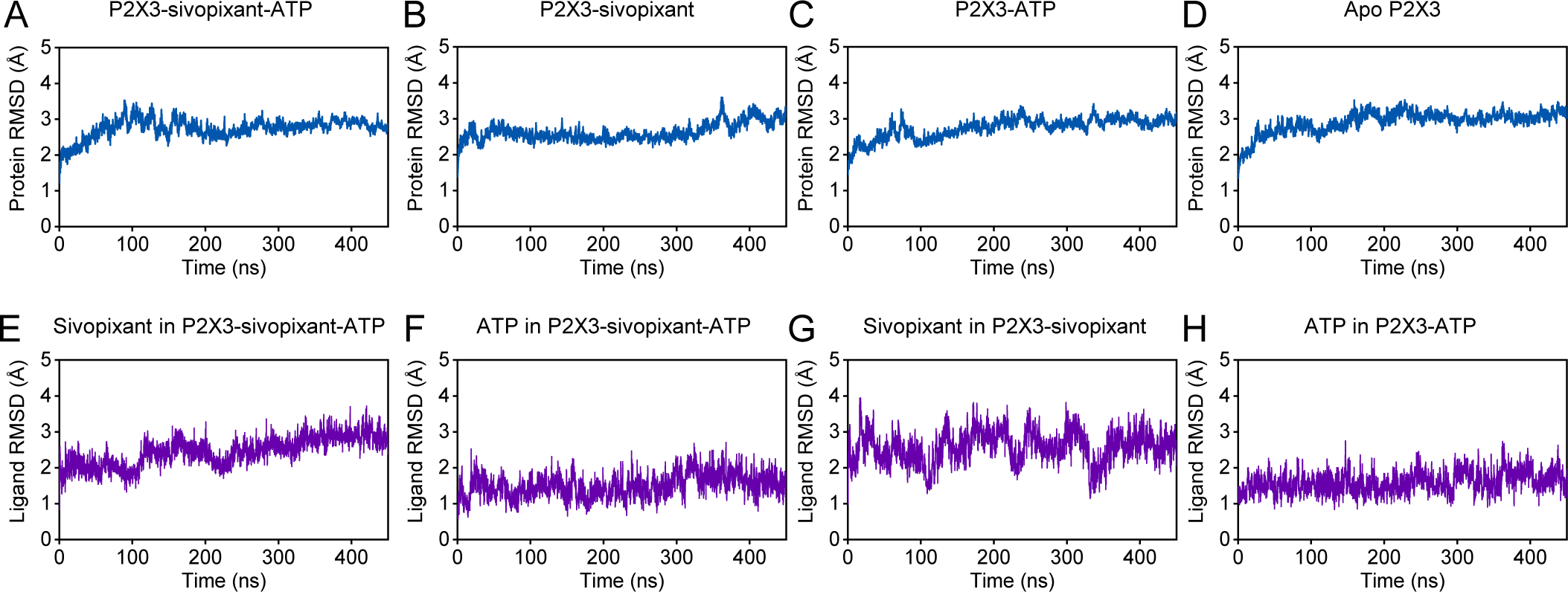

Supplement: S7 Fig — (A–H) MD simulations using the sivopixant- and ATP-bound structure with both retained (A, E, F), ATP deleted (B, G), sivopixant deleted (C, H), and both deleted (D) as starting models. The plots of the root mean square deviations (RMSDs) for Cα atoms (A–D) and the RMSD values of the atoms in sivopixant (E, G) and ATP (F, H). The data underlying this figure can be found in S1 Data. (TIF) [file pbio.3003777.s007.tif]

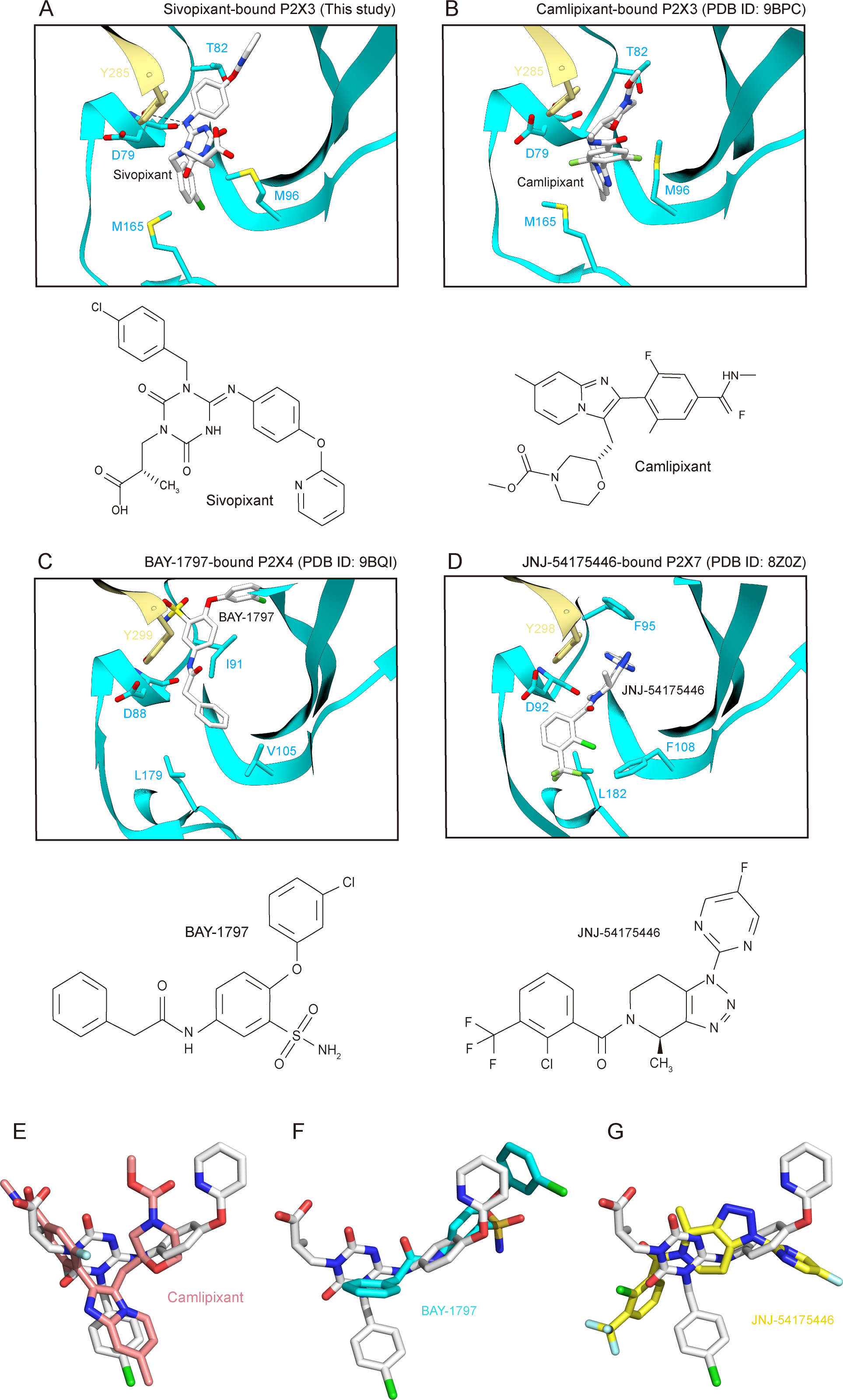

Supplement: S8 Fig — (A–D) Close-up views of the ligand binding sites of the sivopixant- and ATP-bound P2X3 receptor structure (this study) (A), the camlipixant-bound P2X3 receptor structure (PDB ID: 9BPC) (B), the BAY-1797-bound P2X4 receptor structure (PDB ID: 9BQI) (C), and the JNJ-54175446-bound P2X7 receptor structure (PDB ID: 8Z0Z) (D). The chemical structures of each compound are also shown. (E–G) The camlipixant-bound P2X3 (E), BAY-1797-bound P2X4 (F), and JNJ-54175446-bound P2X7 (G) receptor structures were superposed onto the sivopixant- and ATP-bound P2X3 receptor structure, and close-up views of each compound are shown in stick representation together with sivopixant. (TIF) [file pbio.3003777.s008.tif]
