# Supplementary material for: Structure of the human P2X3 receptor reveals the basis for subtype-selective inhibition by sivopixant
Source: PLoS Biol. 2026 Apr 22;24(4):e3003777. doi: 10.1371/journal.pbio.3003777 (PMC13132459; doi:10.1371/journal.pbio.3003777)
Supplement: S1 Table — (PDF) [file pbio.3003777.s011.pdf]

S1 Table. Cryo-EM data collection, refinement and validation statistics.

	P2X3 with Sivopixant and ATP (EMDB:EMD-67624)(PDB: 21FG)	P2X3 with ATP (EMDB:EMD-67603) (PDB: 21DX)
**Data collection and processing**		
Magnification	130000×	130000×
Voltage (kV)	300	300
Electron exposure (e–/Å^2)	40	40
Defocus range (μm)	-1.2 to -1.8	-1.2 to -1.8
Pixel size (Å)	0.959	0.959
Symmetry imposed	C3	C3
Initial particle images (no.)	8747713	8747713
Final particle images (no.)	46550	71855
Map resolution (Å)	3.34	2.95
FSC threshold	0.143	0.143
Map resolution range (Å)	2.07 to 26.15	2.09 to 33.14
**Refinement**		
Initial model used (PDB code)	5SVJ	5SVL
Model resolution (Å)	3.34	2.95
FSC threshold	0.143	0.143
Model resolution range (Å)	2.07 to 26.15	2.092 to 33.142
Map sharpening *B* factor (Å^2)	-83.6	-85.0
Model composition		
Non-hydrogen atoms	7521	7764
Protein residues	981	984
Ligands	SIV: 3; ATP: 3; NAG: 9	NAG: 9; ATP: 3
B factors (Å^2)		
Protein	110.01	106.86
Ligand	115.99	101.18
R.m.s. deviations		
Bond lengths (Å)	0.003	0.002
Bond angles (°)	0.582	0.512
Validation		
MolProbity score	1.52	1.46
Clashscore	3.88	3.73
Poor rotamers (%)	0.00	0.74
Ramachandran plot		
Favored (%)	95.08	95.71
Allowed (%)	4.92	4.29
Disallowed (%)	0	0
