# Supplementary material for: Structure of the human P2X3 receptor reveals the basis for subtype-selective inhibition by sivopixant
Source: PLoS Biol. 2026 Apr 22;24(4):e3003777. doi: 10.1371/journal.pbio.3003777 (PMC13132459; doi:10.1371/journal.pbio.3003777)
Supplement: Attachment — Submitted filename: P2X3_Responses-to-Reviews-rev1.pdf [file pbio.3003777.s015.pdf]

Editorial and data-related requests:

(A) We routinely suggest changes to titles to ensure maximum accessibility for a broad, non-specialist readership. In this case, we would suggest a minor edit to the title, as follows. Please ensure you change both the manuscript file and the online submission system, as they need to match for final acceptance: "Structure of the human PX23 receptor reveals the basis for subtype-selective inhibition by sivopixant"

According to this comment, we have corrected the title in both the manuscript file and the online submission system.

(B) You may be aware of the PLOS Data Policy, which requires that all data be made available without restriction: http://journals.plos.org/plosbiology/s/data-availability. For more information, please also see this editorial: http://dx.doi.org/10.1371/journal.pbio.1001797

Note that we do not require all raw data. Rather, we ask that all individual quantitative observations that underlie the data summarized in the figures and results of your paper be made available in one of the following forms:

-Supplementary files (e.g., excel). Please ensure that all data files are uploaded as 'Supporting Information' and are invariably referred to (in the manuscript, figure legends, and the Description field when uploading your files) using the following format verbatim: S1 Data, S2 Data, etc. Multiple panels of a single or even several

figures can be included as multiple sheets in one excel file that is saved using exactly the following convention: S1_Data.xlsx (using an underscore).

We have prepared the requested supplementary data file in accordance with the journal's guidelines. All relevant data have been uploaded as Supporting Information and are referred to as S1 Data. The file is provided as an Excel file named S1_Data.xlsx, with multiple sheets where applicable.

-Deposition in a publicly available repository. Please also provide the accession code or a reviewer link so that we may view your data before publication.

The atomic coordinates of the human P2X3 receptor structures were deposited in the Protein Data Bank under accession codes 21FG (ATP- and sivopixant-bound, closed) [http://doi.org/10.2210/pdb21FG/pdb] and 21DX (ATP-bound, desensitized) [http://doi.org/10.2210/pdb21DX/pdb], respectively. Cryo-EM maps were deposited in the Electron Microscopy Data Bank (EMDB) under accession codes EMD-67624 (ATP- and sivopixant-bound, closed) [https://www.ebi.ac.uk/pdbe/entry/emdb/EMD-67624] and EMD-67603 (ATP-bound, desensitized) [https://www.ebi.ac.uk/pdbe/entry/emdb/EMD-67603], respectively. The raw electrophysiological trace data were deposited in Mendeley Data (https://doi.org/10.17632/crwytdnsdt).

Regardless of the method selected, please ensure that you provide the individual numerical values that underlie the summary data displayed in the following figure panels as they are essential for readers to assess your analysis and to reproduce it: Figure 3B, 3D, 3F, 5B, 5D, 7B, S5A-H

All individual numerical values underlying the summary data for Fig 3B, 3D, 3F, new 5C (old Fig 5B), new Fig 5E (old Fig 5D), Fig 7B-7E, new S7A-H Fig (old S5A-H Fig), as well as new S6 Fig, are provided in S1_Data.xlsx.

NOTE: the numerical data provided should include all replicates AND the way in which the plotted mean and errors were derived (it should not present only the mean/average values).

(C) Thank you for providing the structural data in the PDB (21FG, 21DX) and EMDB (EMD-67624, EMD-67603) databases. However, we note that the data is currently on hold for release. We ask that you please make the structures publicly available at this stage before publication.

We have already requested the release of the PDB and EMDB entries, and the release date is April 1, 2026.

(D) Please also ensure that each of the relevant figure legends in your manuscript

include information on *WHERE THE UNDERLYING DATA CAN BE FOUND*, and ensure your supplemental data file/s has a legend.

We have revised the manuscript accordingly. The relevant figure legends now clearly indicate where the underlying data can be found, and each supplementary information file has a legend.

(E) Per journal policy, if you have generated any custom code during the course of this investigation, please make it available without restrictions. Please ensure that the code is sufficiently well documented and reusable, and that your Data Statement in the Editorial Manager submission system accurately describes where your code can be found. More information on our Code Policy, what and how to share can be found here: https://journals.plos.org/plosbiology/s/code-availability

We did not generate any custom code during the course of this study. Therefore, there is no original code to share.

(F) Please ensure that your Data Statement in the submission system accurately describes where your data can be found and is in final format, as it will be published as written there.

We have updated the Data Statement in the submission system to accurately describe

where the data can be found, and it is now in its final format as required.

Journal Requirements:

1) Please provide separate figure files in .tif or .eps format. For more information about figure files please see our guidelines:

We have prepared and uploaded all figure files separately in the required .tif formats according to the journal guidelines.

2) We have noticed that you have uploaded Supporting Information files, but you have not included a list of legends. Please add a full list of legends for your Supporting Information files after the references list.

We have added a full list of legends for all Supporting Information files after the references section in the revised manuscript.

3) We do not publish any copyright or trademark symbols that usually accompany proprietary names, eg ©, ®, ™ (e.g. next to drug or reagent names). Please remove all instances of trademark/copyright symbols throughout the text, including:™ on page 18.

We have removed the trademark symbol from the text, including the instance on page 18,

in the revised manuscript.

REVIEWS:

Reviewer #1: This is an interesting and high-quality study on the mechanism of action of a negative allosteric regulator of ATP-activated P2X3 receptor channels. Here the authors solve a cryo-EM structure of P2X3 receptors with sivopixant bound and carefully interrogate the impact of mutations in the binding site on the function effects of the compound on activation by ATP. They identify a number of residues that are critical for the allosteric actions of sivopixant and that help to rationalize the improved subtype selectivity of the drug. The authors also make several gain of function mutants to make P2X1 and P2X2 that greatly strengthen our understanding of the subtype selectivity. Finally, the authors use their structure together with previously available structures to provide a rationale for how sivopixant inhibits opening of P2X3 by ATP. I have only minor comments.

We thank the reviewer for the positive and encouraging evaluation of our work. We are also sincerely grateful for the time and effort devoted to reviewing our manuscript and for the thoughtful and constructive comments. We have carefully addressed all of the comments and provide detailed point-by-point responses below.

1) I would encourage the author to remake most of the structural figures as the colors are so intense that seeing where either ATP or sivopixant binds and the surrounding

interactions are very difficult for the reader to see. I would also like to see density not only for the drug, but for the surrounding residues in the protein so the quality of the maps could be better evaluated.

Thank you for this helpful suggestion. We have revised all structural figures to use less intense colors so that the sivopixant/ATP binding sites and the surrounding interactions can be seen more clearly (Figs 1, 2, 4, 6, 7, S4, S5, and S8). We have also added figures showing the cryo-EM density for the surrounding residues in the sivopixant/ATP binding sites (S3A and S3B Fig).

2) Line 114 should cite Fig. 1C, I believe

We have corrected this (Page 6, Line 119).

3) Line 160 should cite several other figure panels I beleive

We have added citations to Figs 2A and 4C in addition to Fig 2C (Page 8, Line 167)

Reviewer #2: The manuscript by Zhixuan Zhao and colleagues investigates the structural basis for negative allosteric modulation of the human P2X3 receptor by the modulator sivopixant. This includes determining cryo-EM structures of P2X3 bound with ATP and a co-bound ATP and sivopixant structure. Structural findings are

validated through mutagenesis and electrophysiology studies, which include gain-of-function mutations to help understand the selectivity of sivopixant along with molecular dynamic simulations. Overall, this is an extremely well written and well-presented manuscript. Nearly, all the concerns I had were addressed throughout the manuscript, which was refreshing to see.

The overall findings of this work point towards a subtle mechanism of negative allosteric modulation the include dynamic movements of the upper body of the ECD. I believe these findings would be of general interest for those in the P2X biology field and those involved in understanding allosteric modulation of proteins by ligands.

We thank the reviewer for the positive and encouraging evaluation of our work. We are also sincerely grateful for the time and effort devoted to reviewing our manuscript and for the thoughtful and constructive comments. We have carefully addressed all of the comments and provide detailed point-by-point responses below.

Minor concerns.

**** My only real suggestion might be the inclusion of a supplemental figure that shows the cryo-EM map around these regions of difference (in the upper body) to show that there is sufficient data to support the molecular modelling to allow a fair the comparison of structures.***

To follow this comment, we have added a supplemental figure showing the cryo-EM

density map around the regions in the upper body domain to support the molecular modeling and facilitate a fair comparison between the structures (S3C and S3D Fig).

**** Did the authors try camlipixant at the hP2X2-GOF (while I don't think is necessary to perform, it felt like an odd omission).***

We did test camlipixant on the hP2X2-GOF mutant, but did not observe a significant effect even at 10 μM compound concentration. We have now added these data and the corresponding description to the revised manuscript (S6 Fig) (Lines 224-225).

**** Do the authors know how the binding affinity / potency of sivopixant changes based on the presence of absence of ATP? For example, is ATP required for sivopixant binding, and/or does the result in higher or lower potency for sivopixant?***

Although we did not directly measure the binding affinity of sivopixant, our whole-cell patch-clamp data showed that the potency of sivopixant was independent of ATP concentration (Figs 3A and 3B), suggesting that its interaction with the hP2X3 receptor is independent of ATP concentration. We have added some additional description to the revised manuscript (Page 6, Line 128). In addition, in our MD simulations, sivopixant remained stably bound even after ATP removal, supporting the idea that ATP is not essential for sivopixant binding (S7 Fig).

Reviewer #3: This is a valuable, well performed and well presented study. It will be useful for understanding the P2X3 selectivity of NAMs versus the P2X2/X3 heteromeric receptor.

We thank the reviewer for the positive and encouraging evaluation of our work. We are also sincerely grateful for the time and effort devoted to reviewing our manuscript and for the thoughtful and constructive comments. We have carefully addressed all of the minor comments and provide detailed point-by-point responses below.

There are a number of smalle points to be addressed.

1. Title: is too general. Add: "by Sivopixant".

Structure of the human P2X3 receptor reveals the basis for subtype-selective inhibition by sivopixant

We agree that the original title was too general. In response, we have revised the title to: “Structure of the human P2X3 receptor reveals the basis for subtype-selective inhibition by sivopixant.”

2. It is disturbing that the authors use "P2X3" as an abbreviation for P2X3 receptor in many places. If you want to use an abbreviations, use P2X3R (and define it before use). This has to be corrected throughout.

Thank you for this helpful comment. In response, we have revised the manuscript throughout so that instances where "P2X3" or other P2X subtype designations were used alone to refer to the receptor have been corrected to the corresponding receptor term(s) (for example, "P2X3 receptor" or "P2X3 receptors"), as appropriate.

3. p. 4, lines 79-80: For other potential drug targets among the P2XRs, particularly the P2X7R, several NAM-bound structures have been reported. (Change the sentence. The current sentence is an exaggeration).

Following this comment, we revised the sentence to use a more cautious tone (Page 4, Lines 81-82).

4. Please show chemical structures of all discussed compounds, and compare them.

The chemical structures of all discussed compounds are now shown in S8A-D Fig (old S6A-D Fig). In addition, a structural comparison of these compounds has been added in S8E-G Fig and discussed in the revised manuscript (Page 14, Lines 294-302).

5. p. 5, line 92: There are more structures with ATP, e.g. Shi et al. Sci. Adv. 2024; Kim et al., JMC 2024; etc.

We appreciate this helpful comment. In response, we examined *Shi et al., Sci. Adv.* and

found that the reported structures were either a binary complex with a NAM (PDB ID: 9BQI) or a binary complex with ATP (PDB ID: 9C48), but not a ternary complex containing both ATP and a NAM. In contrast, *Kim et al., JMC* reported a complex structure with compound 26a as a ternary complex containing ATP (PDB ID: 9IK1). Notably, because compound 26a binds to the TM domain and stabilizes the desensitized state, its mode of action differs from that of other NAMs, such as sivopixant, which act at the typical allosteric site in the extracellular domain to stabilize the closed state. Based on this, we added a citation to *Kim et al., JMC* and revised the text accordingly (Page 5, Lines 93-99).

6. p. 5, line 108: Fig. 2 is wrong here.

We removed it in the revised manuscript (Page 6, Line 115).

7. p.5, line 92: Several ATP/NAM-bound structures have been published, e.g. Shi et al., 2024; Kim et al., 2024 ...

This comment appears to raise the same issue as Comment 5, and we have addressed it as described above.

Various points

p. 6, line 130: …"sivopixants binds at each interface of the homotrimer…" (please

correct)

We have revised the sentence accordingly (Page 7, Line 137).

p. 7. Line 137 typo Sivopixant

We have corrected the typo (Page 7, Line 144).

p. 7, line 142, 143: side chain Asp79 (insert side chain)

We have revised the sentence accordingly (Page 7, Lines 149-151).

p. 7, line 150: citation missing ("our previous…")

We have added the citation (Page 8, Line 158).

p. 8, line 160: not "commonly", only found in some…

We have revised the sentence accordingly (Page 8, Line 167).

p. 10: Is there an explanation why the GoF mutant of P2X1R changes behavior towards sivopixant and only marginally for camlipixant? Compare with published structure,

Thach et al., JBC 2025).

Again, it would be helpful to show all chemical structures of the antagonists and compare them and their interactions.

Based on Thach et al. (JBC 2025), we added to the discussion (Page 11, Lines 227-234) that Tyr70, Arg73, and Met75 in the human P2X3 receptor, which did not appear to be involved in sivopixant binding or sensitivity in our structural and mutational analyses, are involved in camlipixant binding. This difference may partly explain why the gain-of-function mutant designed on the basis of the sivopixant-bound structure showed only a partial increase in sensitivity to camlipixant.

In addition, the addition of the chemical structures and their comparison has already been addressed in our response to this reviewer's earlier comment (S8 Fig) (Page 14, Lines 294-302).

p. 11: ATP-bound vs. non-ATP-bound structures with NAM: Do you expect that ATP changes the interactions?

This question is essentially the same as one of the comments raised by Reviewer 2, and our response is therefore also the same. Although we did not directly measure the binding affinity of sivopixant, our whole-cell patch-clamp data showed that the potency of sivopixant was independent of ATP concentration (Figs 3A and 3B), suggesting that its interaction with the hP2X3 receptor is independent of ATP concentration. We have added

some additional description to the revised manuscript (Page 6, Line 128). In addition, in our MD simulations, sivopixant remained stably bound even after ATP removal, supporting the idea that ATP is not essential for sivopixant binding (S7 Fig).

p. 14, line 303: The clinical candidates are already optimized! How could they be further optimized? Any suggestions?

Following this comment, we removed the word "optimized" and revised the sentence accordingly to avoid overstatement (Page 15, Line 328).

p. 15 line 330: Please check the procedure. Is it just dialysis or cleavage? Volume to perform dialysis?

TEV protease was added to the purified GFP-fusion protein and that the cleavage reaction was carried out during dialysis. The volume of the dialysis buffer was 1 liter. To clarify, we revised the text accordingly (Page 17, Lines 354-356).

p. 23, line 488: some mutants (only the mutants that show a significant change are shown).

We revised it accordingly (Page 24, Line 520).

p. 24 Figure 5: add the mutantions introduced for GoF.

In response to this comment, we added a new Fig 5A showing the introduced mutations. Accordingly, the original Fig 5A-D have been renumbered as Fig 5B-E.

p. 25, Fig. 7: how many replicates?

Regarding this point, a single MD run was performed for each of the four conditions, and we have added this information to the figure legend (Page 27, Lines 573-574).

References: Many references cited in the Introduction are rather old. Please update.

To address this comment, we replaced old review articles published more than 10 years ago with more recent ones, while retaining old original articles that are historically important, such as those proposing purinergic signaling and those reporting the identification of P2X receptors. Specifically, the following references were updated:

Ref. 3: North, R. A. (2016) was replaced with Illes P, Müller CE, Jacobson KA, et al. (2021) (PMID: 33125712).

Ref. 4: North, R. A. (2002) was replaced with Oken AC, Krishnamurthy I, Savage JC, et

al. (2022) (PMID: 35784697).

Ref. 7: Burnstock, G., Nistri, A., Khakh, B. S. & Giniatullin, R. (2014) was replaced with Guo Y, Mao T, Fang Y, et al. (2025) (PMID: 38565403).

Ref. 9: Burnstock, G. (2000) was replaced with Spinaci A, Buccioni M, Dal Ben D, et al. (2021) (PMID: 33927627).

Ref. 12: North, R. A. (2004) was replaced with Krajewski JL. (2020) (PMID: 33009633).

Formatting of many of the references requires editing/correction.

We carefully checked the formatting of the references and corrected them throughout the manuscript.

Figures: Consider optimizing the coloring schemes.

We also received similar comment from other reviewers and have revised all structure figures using a softer color scheme to improve its clarity (Figs 1, 2, 4, 6, 7, S4, S5, and S8).
